# Genetic inbreeding depression load for morphological traits and defects in the Pura Raza Española horse

**DOI:** 10.1186/s12711-020-00582-2

**Published:** 2020-10-20

**Authors:** Julia Poyato-Bonilla, Davinia I. Perdomo-González, María J. Sánchez-Guerrero, Luis Varona, Antonio Molina, Joaquim Casellas, Mercedes Valera

**Affiliations:** 1 Departamento de Ciencias Agroforestales, ETSIA, Universidad de Sevilla, Ctra. Utrera km. 1, 41013 Sevilla, Spain; 2 Departamento de Anatomía Embriología Y Genética Animal, Instituto Agroalimentario de Aragón (IA2), Universidad de Zaragoza, Zaragoza, 50013 Spain; 3 Departamento de Genética, Universidad de Córdoba, Córdoba, 14071 Spain; 4 Departament de Ciència Animal I Dels Aliments, Universitat Autònoma de Barcelona, Bellaterra, 08193 Spain

## Abstract

**Background:**

Inbreeding is caused by mating between related individuals and is associated with reduced fitness and performance (inbreeding depression). Several studies have detected heterogeneity in inbreeding depression among founder individuals. Recently, a procedure was developed to predict hidden inbreeding depression load that is associated with founders using the Mendelian sampling of non-founders. The objectives of this study were to: (1) analyse the population structure and general inbreeding, and (2) test this recent approach for predicting hidden inbreeding depression load for four morphological traits and two morphology defects in the Pura Raza Española (PRE) horse breed.

**Results:**

The regression coefficients that were calculated between trait performances and inbreeding coefficients demonstrated the existence of inbreeding depression. In total, 58,772,533 partial inbreeding coefficients (*F*_ij_) were estimated for the whole PRE population (328,706 horses). We selected the descendants of horses with a *F*_ij_ ≥ 6.25% that contributed to at least four offspring and for which morphological traits were measured for the subsequent analysis of inbreeding depression load (639 horses). A pedigree was generated with the last five generations (5026 animals) used as the reference population (average inbreeding coefficient of 8.39% and average relatedness coefficient of 10.76%). Heritability estimates ranged from 0.08 (cresty neck) to 0.80 (height at withers), whereas inbreeding depression load ratios ranged from 0.01 (knock knee) to 0.40 (length of shoulder), for an inbreeding coefficient of 10%. Most of the correlations between additive and inbreeding depression load genetic values and correlations between inbreeding depression load genetic values for the different traits were positive or near 0.

**Conclusions:**

Although the average inbreeding depression loads presented negative values, a certain percentage of the animals showed neutral or even positive values. Thus, high levels of inbreeding do not always lead to a decrease in mean phenotypic value or an increase in morphological defects. Hence, individual inbreeding depression loads could be used as a tool to select the most appropriate breeding animals. The possibility of selecting horses that have a high genetic value and are more resistant to the deleterious effects of inbreeding should help improve selection outcomes.

## Background

Inbreeding is a well-known biological phenomenon that derives from the mating of related individuals. Offspring from inbred mating present a higher degree of homozygosity across the genome, which results in unmasking recessive deleterious mutations or losing the advantage from alleles with heterozygous superiority [1]. In domestic species, animals are selected for breeding with the most outstanding individuals being more frequently used, which leads to an unequal contribution of a limited number of breeding animals to the next generation. Thus, stock breeders face increases in inbreeding in their populations. Such increases are typically associated with reduction in animal fitness and, in turn, with the loss of performance, mainly for reproductive capacity or physiological efficiency, but all traits can be affected [2].

The effects of inbreeding depression have been frequently analysed, and it is assumed that inbreeding coefficients ($F$) calculated from the pedigree are linearly related to phenotypic values of a trait in the absence of epistasis. However, this approach does not take several factors into account: (1) the fact that the offspring of different founders may be differently affected by an uneven distribution of the recessive genetic load, (2) that founder lines are exposed to variable selection pressures, or (3) that a large number of loci are involved in inbreeding depression phenomena [3, 4]. For example, siblings do not necessary inherit the same alleles from their parents and the inbreeding coefficient of any two animals does not necessarily originate from the same ancestor [5]. The possibility of computing partial inbreeding coefficients (those related to alleles transmitted by a specific ancestor) provides a way of testing the genetic load that is distributed heterogeneously among founder genomes. This variability in inbreeding depression has been confirmed within ancestral lineages of Drosophila [6] and in sire families in dairy cattle [7], sheep [8] and beef cattle [9, 10], mainly for growth or milk traits. The only work published in horse [11] focused on racing performance and studied the cumulative earnings, earnings per start, career length, total number of starts and winning strike rate as variables. These studies revealed a wide range of neutral, negative and even positive effects of founder-specific inbreeding in livestock, although complete inbreeding depression tends to have deleterious effects on the traits measured. This explains that some individuals will exhibit deleterious effects from inbreeding whereas others, with the same levels of $F,$ will not.

Variation in inbreeding depression among lineages may have consequences for the genetic management of breeds. Knowledge of the detrimental or beneficial effects of founder alleles for traits of interest allows breeders to select the most appropriate breeding animal. Casellas [5] proposed a model, which describes inbreeding depression load by using founders and assuming the additive nature of the individual inbreeding depression load, while Varona et al. [10] added the Mendelian sampling of non-founders in order to estimate this value.

The PRE or Andalusian horse is one of the oldest European horse breeds and the most well known in the Iberian Peninsula, with almost 250,000 active individuals across 60 countries; it is also the second breed in the World Breeding Federation for Sport Horses (WBFSH) with the highest census. Today, this breed is used mainly as a leisure horse and in equestrian sports particularly in dressage. Although the population of PRE horses is large, genealogical analyses have demonstrated that inbreeding levels are significantly high [12, 13] because the number of its founders is small, selection for desired traits is carried out in a closed population, and inbred matings are performed on the studs.

The PRE Breeding Program was initiated in 2003 and has set as its main objectives the improvement of the breed’s morphology, conformation and functionality [14]. The economic value of a PRE horse, which is mostly used as a riding horse or in the discipline of dressage, depends highly on its body measurements and moving ability [15]. In addition, almost all the individuals pass a basic morphological test, which is mandatory if the breeder wants to use them for reproduction. Since 2012, apart from zoometrics, the classification of some of the variables of horse morphology has followed a linear system. Besides, the population of PRE horses is checked for a range of defects and diseases related to morphology, such as cresty neck and melanomas.

Although previous studies have reported the existence of significant inbreeding depression for body measurements in this breed [16] and a study in horses investigated the effects of founder-specific inbreeding depression [8], there is no published research on the inbreeding depression load that considers common ancestors in horses, or that analyses morphological traits or several traits jointly in any species. The first aim of our study was to analyse the structure of the PRE population and its inbreeding level. The second objective was to perform, for the first time in an equine breed, a genetic analysis of the potential inbreeding depression load for four morphological traits and two morphological defects in animals that are frequently used for breeding. These breeding animals are responsible for increased homozygosity and therefore for inbreeding depression load, with serious implications for the Breeding Programme in PRE breed.

## Methods

### Dataset

#### Pura Raza Española horse population

Pedigree data were obtained from the Asociación Nacional de Criadores de Caballos de Pura Raza Española (ANCCE) studbook, for 328,706 animals (160,640 males and 168,066 females) born from the early 1900s to 2018, among which 99,400 horses have been used as breeders (23,530 stallions and 75,870 mares).

The partial inbreeding coefficients from Mendelian decomposition were calculated by García-Cortés et al. [17] for all the individuals in the population, and 58,772,533 coefficients were derived from 10,244 ancestors.

In this study, we used two morphological traits that are not under selection, height of withers (HofW) and scapular-ischial length (SIL), and two morphological traits under selection, height at withers (HatW) and length of shoulder (LofS). Two morphological defects under selection were also studied: knock knee (KK) and cresty neck (CrN). HatW and LofS are related to biokinematics in trot and dressage performance, respectively, and KK and CrN are disqualifying defects for registration as breeding animals in the PRE breed studbook [see Additional file 1 Figure S1]. The morphological traits were measured in cm.

Morphological data for 52,503 stallions and 81,556 mares were used. The number of animals with records on morphological traits and defects is in Table 1 and ranged from 2536 stallions and 3763 mares for CrN to 49,165 males and 77,351 females for SIL.

**Table 1:** Descriptive statistics and means for four morphological traits and two morphological defects analysed in the PRE population for males and females

Body traits	Males			Females		
	n	Mean (SD)	CV%	n	Mean (SD)	CV%
Height of withers (cm)	10,533	8.40 (1.88)^a^	22.35	18,343	8.18 (1.92)^b^	23.51
Height at withers (cm)	9028	162.27 (4.68)^a^	2.88	17,155	159.53 (4.66)^b^	2.92
Length of shoulder (cm)	23,159	66.91 (3.74)^a^	5.59	40,656	66.09 (3.97)^b^	6.01
Scapular-ischial length (cm)	49,165	159.38 (5.22)^a^	3.28	77,351	159.01 (5.30)^b^	3.34
Knock knee^c^	3556	2.22 (0.45)^a^	19.61	5340	2.23 (0.44)^a^	19.83
Cresty neck^c^	1293	3.15 (1.43)^a^	45.35	1858	3.05 (1.32)^a^	43.33

*n* number of records, *SD* standard deviation and *CV* coefficient of variation

^a,b^ Different superscript letters indicate significant differences between genders (P < 0.05)

^c^Only animals that present the defects were considered (classes > 1 on the scale)

#### Reference population

In order to study the inbreeding depression load, only the ancestors that contribute with a partial inbreeding coefficient higher than 6.25% to four or more offspring with morphological data available were considered (639 horses). From these animals, a pedigree with the last five generations was generated (5026 animals: 1662 stallions and 3364 mares). The final database included records for the selected horse offspring, i.e. 2732 animals (919 males and 1813 females) born between 1977 and 2013.

### Statistical and genetic variability analysis

Descriptive statistics of the morphological traits, the Tukey HSD test (for mean comparison tests), regression coefficients and Pearson’s correlations were performed using the SPSS software for Windows (version 25.0, IBM statistics data editor, IBM Corp. Released 2017. IBM SPSS Statistics for Windows, Version 25.0. Armonk, NY: IBM Corp.).

The Endog v4.8 program was used to carry out all the genealogical analyses [18]. The probability of gene origin and the genetic variability were analysed in the whole population (328,706 animals), in the population of 264,084 active animals (i.e. from the last two generations with a generation interval of 10 years [13]), and in the reference PRE population (5026 animals) in order to shed light on the status of the genetic links between individuals, the level of inbreeding, the kinship between animals and the representativeness of the sample.

The probability that an individual possesses two identity-by-descendent (IBD) alleles at a randomly chosen locus was measured by the inbreeding coefficient ($F$) [19], which was computed following the methods described by Meuwissen and Luo [20]. Inbreeding at the 3rd (${F}_{3}$) and 6th generation (${F}_{6}$) were also computed. The increase in inbreeding ($\Delta F$) was calculated by the formula

$$\Delta F=({F}_{t}- {F}_{t-1})/(1-{F}_{t-1}),$$

where ${F}_{t}$ is the average inbreeding at generation $t$ [18].

To study the probability that a randomly chosen allele from the population belongs to an individual, the average relatedness coefficient ($\mathrm{AR}$) was computed. This is calculated as the average of the coefficients that integrate the row from the individual in the numerator relationship matrix and it considers, simultaneously, the inbreeding and coancestry coefficients, thus it can be interpreted as the representation of the animal in the whole pedigree [21, 22].

For each individual in the pedigree, the following values were computed: the maximum number of generations, the complete generations and the equivalent complete generations. The maximum number of generations is defined as the oldest generation for which all the ancestors are known. Ancestors with unknown parents were considered as founders (last generation). Complete generations value is the number of generations that separates the individual from its furthest ancestor. The equivalent complete generations value is calculated as the sum of the terms across all known ancestors, or the sum of (1/2)^*n*^ where *n* is the number of generations separating the individual to each known ancestor [23]. According to the literature [13], the average generation interval in PRE is approximately 10 years, which is the value used throughout this study.

Genetic variability was studied across the number of founders, the number of ancestors, and the effective number of founders (${F}_{e}$), defined as the number of equally contributing founders that would be expected to produce the same genetic diversity as in the population under study. We also studied the effective number of ancestors (${F}_{a}$), in other words, the minimum number of ancestors, not necessarily founders, that explain the complete genetic diversity of a population; and the number of equivalent founders (${F}_{eq}$), i.e. the number of founders that would be expected to produce the same genetic diversity as in the population under study if the founders were equally represented and no loss of alleles occurred.

### Inbreeding decomposition and the inbreeding depression linear model

The data for each morphological trait ($\mathbf{y}$) was analysed under a linear model. This model was proposed by Casellas [5], in which the phenotypic values are explained by two random genetic effects, the standard breeding value and the inbreeding depression load generated by its ancestors. At a later stage, this model was reformulated by Varona et al. [10] to reflect the additive nature of the individual inbreeding depression load ($\mathbf{i}$). This reparameterization allowed a model reformulation:

$$\mathbf{Y}=\mathbf{X}\mathbf{b}+\mathbf{Z}\mathbf{u}+\mathbf{K}\mathbf{i}+\mathbf{e},$$

where: $$\mathbf{u}\sim \mathrm{N}({\mathbf{0}},{\mathbf{A}\upsigma }_{\mathrm{u}}^{2}),$$ $$\mathbf{i}\sim \mathrm{N}({\mathbf{0}},{\mathbf{A}\upsigma }_{\mathrm{i}}^{2});$$ and $$\mathbf{e}\sim \mathrm{N}({\mathbf{0}},\mathbf{I}{\upsigma }_{\mathrm{e}}^{2}).$$

This model takes the systematic effects ($\mathbf{b}),$ infinitesimal additive genetic contributions ($\mathbf{u}),$ individual inbreeding depression load effects (**i**) and residual terms ($\mathbf{e}$) into account. $\mathbf{A}$ is the numerator relationship matrix, and ${\upsigma }_{\mathrm{u}}^{2}$, ${\upsigma }_{\mathrm{i}}^{2}$ and ${\upsigma }_{\mathrm{e}}^{2}$ are the associated variance components. $\mathbf{X}$ and $\mathbf{Z}$ are incidence matrices of systematic effects and additive genetic contributions, respectively, and $\mathbf{K}=\mathbf{T}(\mathbf{I}-\mathbf{P})$. $\mathbf{T}$ is a lower triangular matrix in which each non-zero element is a partial inbreeding coefficient obtained by Mendelian decomposition of inbreeding following the procedure used by García-Cortés et al. [17], which links the phenotype of an inbred individual to the ancestor causing inbreeding. $\mathbf{P}$ is a projection matrix with a 0 diagonal and 0.5 in the elements that link an individual to its sire and dam. More specifically, $\mathbf{b}$ includes the sex of the horse (2 levels), its age on the day the morphological evaluation is performed (6 levels) and the geographic stud zone, with 35 levels for all traits except for length of shoulder (37 levels) and scapular-ischial length (39 levels).

The MCMC model was implemented using ad hoc software written in FORTRAN90 (10) and one single chain of 525,000 samples and a burning period of 25,000 were used.

## Results

Table 1 describes the statistics for each morphological trait and defect analysed separately for males and females. The total number of animals analysed for morphology traits ranged from 126,516 for SIL to 26,183 for HatW, whereas the number of animals analysed for morphological defects was 8896 for KK and 3151 for CrN. Significant differences in the measures for all morphological traits (measured in cm) were found between genders, and in all cases, mean values were higher in males than females. No differences between genders were observed for the morphological defects. These results show that CrN is the most common defect in the PRE breed in both genders with mean values of 3.0, whereas KK reaches a mean value of 2.0, which are moderate and light levels of the defects, respectively. Regarding the coefficients of variation, CrN stands out for its wide variability.

Table 2 shows that the number of maximum generations ranged from 14 to 18 between the reference, active and total populations, and the equivalent and complete generation results ranged from 7.8 to 9.9, and from 4.7 to 5.9, respectively, with the active population and analysed population having the largest and smallest number of known generations, respectively.

**Table 2:** Probability of gene origin and genetic variability in the populations of PRE horses

	Total population	Active population	Reference population
n	328,706	264,084	5026
Maximum generations	17.14	17.96	14.52
Equivalent generations	9.44	9.93	7.81
Complete generations	5.64	5.94	4.67
Founders	1056	428	344
${F}_{e}$	33	33	36
${F}_{eq}$	871	335	281
Ancestors	1026	301	222
${F}_{a}$	19	19	21
$F$	7.51%	7.43%	8.39%
$F>0$	98.74%	99.98%	96.62%
${F}_{6}$	3.20%	2.75%	6.08%
${F}_{3}$	1.22%	1.06%	3.13%
$\Delta F$	0.96%	0.88%	1.33%
$\mathrm{AR}$	11.31%	11.37%	10.76%

*n* number of animals, ${F}_{e}$ effective number of founders, ${F}_{eq}$ number of equivalent founders, ${F}_{a}$ effective number of ancestors, $F$ classical inbreeding coefficient, $F>0$: % of animals with an $F$ higher than 0, ${F}_{6}$ inbreeding at 6th generation, ${F}_{3}$ recent inbreeding coefficient (at 3rd generation), $\Delta F$ intergenerational average increase in inbreeding, $AR$ average relatedness

Total population comprises the whole Pura Raza Española horse population; active population comprises the last two generations (each generation is 10 years); reference population comprises the last five generations from horses with measured morphological traits who are descendants of horses contributing with a ${F}_{ij}$ ≥ 6.25% in at least four offspring

The number of founders ranged from 344 in the reference population to 1056 in the total population. Regarding effective and equivalent numbers of founders, values ranged from 33 in the total and active populations to 36 in the reference population, and from 281 (reference population) to 871 (total population). The number of ancestors (individuals with known parents that explain the genetic variability of the population) reached 222 in the reference population, 301 in the active population and 1026 in the total population. The total and active populations had an effective number of ancestors of 19 whereas for the reference population it was 21.

Results for inbreeding coefficients analysed with classical $F$, and recent $F$ (${F}_{3}$ and ${F}_{6}$) and $\Delta F$, in the total, active and reference PRE populations, are also in Table 2. The average values of these parameters are higher in the reference population than in the other two, since the reference animals were intentionally selected from horses that gave them a relatively high ${F}_{ij}$ (≥ 6.25%). The active population, in turn, showed slightly lower values than the total population. However, in the reference population, both the percentage of animals with $F>0$ and the $\mathrm{AR}$ coefficient are very similar but slightly lower to those in the other populations.

Regression coefficients between the traits analysed and inbreeding coefficients ($F$, ${F}_{3}$ and ${F}_{6}$) and the increase in inbreeding ($\Delta F$) are in Table 3. The regression coefficients between the morphological values and $F$ ranged from − 8.12 for SIL to 1.21 for CrN, and were all negative results except for CrN. This can be interpreted as a decline in the phenotypic values of morphological traits and an increase in the prevalence of the defects when there is a higher level of inbreeding. Regarding the regression coefficients with ${F}_{3}$ and ${F}_{6}$, the values became more strongly negative for the morphological traits and increased for the morphological defects as the number of generations increased. According to our results, the regression coefficients between body measurements and $\Delta F$, which reached −71.40 for SIL and 11.23 for CrN, are in line with the inbreeding coefficients (−8.12 and 1.21, respectively), and showed negative values in the case of morphological traits and positive values for CrN. In general, most of the coefficients are significant.

**Table 3:** Inbreeding regression coefficients for the morphological traits and defects analysed in the PRE horse

	${{\varvec{F}}}_{3}$	${{\varvec{F}}}_{6}$	${\varvec{F}}$	$\Delta {\varvec{F}}$
Height of withers	–1.29^*^	–1.43^*^	–1.97^*^	–14.40^*^
Height at withers	0.56	–0.74	–6.64^*^	–60.70^*^
Length of shoulder	–0.36	0.28	–2.76^*^	–14.34^*^
Scapular-ischial length	–5.06^*^	–7.87^*^	–8.12^*^	–71.40^*^
Knock knee***	–0.27	–0.37^*^	–0.24^**^	–2.37^*^
Cresty neck***	–0.63	0.30	1.21^**^	11.23^*^

*Significant at p < 0.01; **significant at p < 0.05; ***only animals that present the defects were considered (classes > 1 in the scale)

${F}_{3}$ inbreeding at 3rd generation, ${F}_{6}$ inbreeding at 6th generation;$F$ global inbreeding coefficient, $\Delta F$ intergenerational average increase in inbreeding

Table 4 shows the heritability estimates, inbreeding depression load ratios and the variances attributed to the direct additive genetic effect, the inbreeding depression load (corresponding with an inbreeding value of 10%), and the residual effect of the analysed traits estimated in the reference population. The heritability estimates were highest for HatW (0.80) and SIL (0.34), which are the two morphological traits that primarily define the height and length of the horse and its proportionality index. For the other traits and defects, they ranged from 0.08 (CrN) to 0.16 (KK).

**Table 4:** Heritabilities, inbreeding load ratios and additive, depression load and residual variances in the reference population

	h^**2**^	d^**2**^	${{\varvec{\upsigma}}}_{\mathbf{a}}$			${{\varvec{\upsigma}}}_{\mathbf{d}}$			${{\varvec{\upsigma}}}_{\mathbf{e}}$		
			Mean (SD)	Median	HPD_**95%**_	Mean (SD)	Median	HPD_**95%**_	Mean (SD)	Median	HPD_**95%**_
Height of withers	0.10	0.26	0.05 (0.03)	0.04	0.00, 0.10	12.08 (12.41)	9.42	0.00, 35.72	0.29 (0.03)	0.29	0.24, 0.35
Height at withers	0.80	0.06	1.84 (0.27)	1.85	1.31, 2.35	12.71 (30.34)	11.90	0.00, 72.92	0.32 (0.19)	0.30	0.00, 0.65
Length of shoulder	0.12	0.40	0.25 (0.08)	0.24	0.10, 0.40	80.80 (57.93)	79.94	0.00, 180.72	1.00 (0.08)	1.00	0.85, 1.15
Scapular-ischial length	0.34	0.30	1.20 (0.11)	1.20	1.00, 1.42	106.54 (46.39)	103.21	18.74, 192.79	1.30 (0.08)	1.30	1.15, 1.45
Knock knee	0.16	0.01	< 0.01 (< 0.01)	0.00	0.00, 0.01	0.01 (0.02)	0.00	0.00, 0.03	0.01 (0.00)	0.01	0.00, 0.02
Cresty neck	0.08	0.04	0.01 (0.01)	0.01	0.00, 0.02	0.25 (0.47)	0.03	0.00, 1.20	0.06 (0.01)	0.07	0.05, 0.08

*h*^*2*^ heritability, *d*^*2*^ inbreeding depression load ratio, ${\sigma }_{a}$ direct additive genetic variance, ${\sigma }_{d}$ inbreeding depression load variance,${\sigma }_{e}$ residual variance, *SD* standard deviation, *HPD*_*95%*_*:* highest posterior density at 95%

Values correspond to a horse with an inbreeding value of 10%

Inbreeding depression load ratios that were calculated for an inbreeding value of 10%, ranged from 0.01 (KK) to 0.40 (LofS). For HofW and LofS, the inbreeding depression load variances values were larger than the direct additive genetic variances. Because of this, the values of the inbreeding depression load surpassed the heritability estimates. Finally, the highest posterior density (HDP) intervals due to the direct additive genetic effect variances (${\upsigma }_{\mathrm{a}}$ HPD_95%_) reach 0 for HofW, KK and CrN, whereas the HDP intervals due to inbreeding depression load variances (${\upsigma }_{\mathrm{d}}$ HPD_95%_) are equal to 0 for all traits except SIL.

**Fig. 1:**
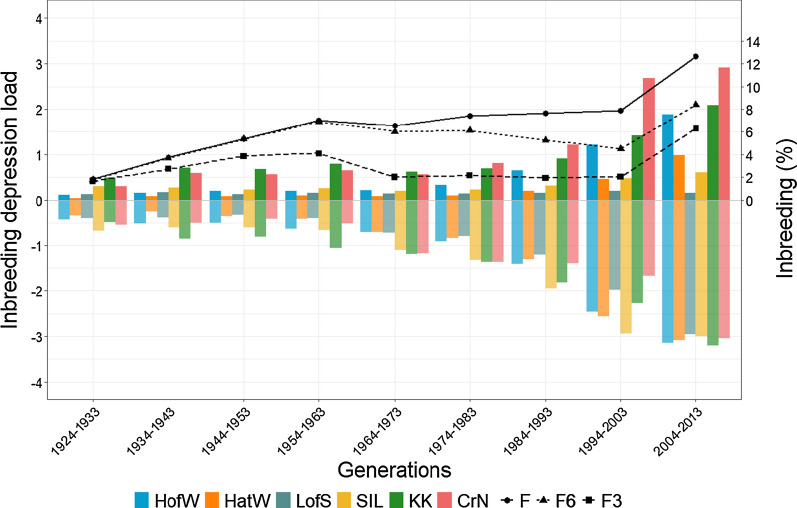
Average inbreeding depression loads for all analysed traits and inbreeding coefficient changes across the last nine generations in the reference population. Positive values are averages for animals with positive inbreeding depression load genetic values. Negative values are averages for animals with negative inbreeding depression load genetic values. *HofW* Height of withers, *HatW* Height at withers, *SIL* Scapular-ischial length, *LofS* Length of shoulder; *KK* Knock knee, *CrN* Cresty neck, $F$ global inbreeding coefficient, ${F}_{6}$ inbreeding at 6th generation, ${F}_{3}$ inbreeding at 3rd generation

Figure 1 shows the variations in the average, positive and negative values of the inbreeding depression load and in the overall and recent inbreeding coefficients at generations 3 and 6 across the last nine generations (last 90 years) in the reference population. Figure 1 shows that the three classical inbreeding parameters ($F$, ${F}_{6}$ and ${F}_{3}$) underwent an initial increase, followed by a stabilization and a final rise in the last two generations. These results reflect the effort made by breeders to contain the increase in inbreeding and the fact that the selected animals for this study had high inbreeding values. However, total average inbreeding depression loads have become, in general, more negative across generations, and the largest increases in negative inbreeding depression loads are accompanied by an upward trend in the classical $F$ value. Even when average inbreeding depression load values for the morphological traits and defects are negative, some animals in the population have positive values. In Fig. 1, the upper part shows average inbreeding depression load for animals with positive values and the lower part shows the average inbreeding depression load for animals with negative values. While the negative values, in general, tend to become more negative across generations, the positive values do not seem to follow an upward trend across generations for all the traits, i.e. some appear to be constant, as for LofS, or to increase slightly, as for SIL and HatW*.*

Finally, Pearson’s correlations between breeding values were estimated as an estimator of genetic correlations between traits [24]. Correlations between the potential inbreeding depression load genetic values (IL) and breeding values (BV) are in Table 5. On the diagonal, the results correspond to the correlations between the variables for the same trait, which ranged from a highly negative value, −0.682 (CrN), to a highly positive value, 0.669 (HofW). Off the diagonal, the correlations between IL and BV for different traits, which were all low. The strongest positive correlation between IL and BV was between IL_HatW and BV_HofW (0.257). Conversely, the strongest negative correlations were between IL_LofS and BV_KK (−0.182), between IL_LofS and BV_CrN (−0.177), and between IL_CrN and BV_HofW (−0.176).

**Table 5:** Pearson’s correlations (± s.d.) between inbreeding depression load genetic values and breeding values in the reference population

	BV_HofW	BV_HatW	BV_LofS	BV_SIL	BV_KK	BV_CrN
IL_HofW	0.669 ± 0.010^*^	0.114 ± 0.014^*^	0.073 ± 0.014^*^	–0.022 ± 0.014	0.122 ± 0.014^*^	0.070 ± 0.014^*^
IL_HatW	0.257 ± 0.014^*^	0.226 ± 0.014^*^	0.020 ± 0.014	–0.024 ± 0.014	–0.157 ± 0.014^*^	0.016 ± 0.014
IL_LofS	0.037 ± 0.014^*^	0.149 ± 0.014^*^	0.356 ± 0.013^*^	–0.048 ± 0.014^*^	–0.182 ± 0.014^*^	–0.177 ± 0.014^*^
IL_SIL	0.166 ± 0.014^*^	0.072 ± 0.014^*^	0.000 ± 0.014	–0.068 ± 0.014^*^	0.031 ± 0.014	–0.084 ± 0.014^*^
IL_KK	–0.073 ± 0.014^*^	0.087 ± 0.014^*^	0.047 ± 0.014^*^	–0.011 ± 0.014	–0.556 ± 0.012^*^	0.080 ± 0.014^*^
IL_CrN	–0.176 ± 0.014^*^	0.019 ± 0.014	0.134 ± 0.014^*^	0.025 ± 0.014	–0.112 ± 0.014^*^	–0.682 ± 0.010^*^

*BV* breeding value, *IL* inbreeding load genetic value, *HofW*

Height of withers, *HatW *height at withers, *LofS* length of shoulder, *SIL* scapular-ischial length, *KK* knock knee, *CrN* cresty neck

*Significant differences p < 0.05

Table 6 shows the Pearson’s correlations between IL, the percentage of males and females in the upper and lower decile of IL compared to the reference PRE population and the percentage of coincident animals between traits in the upper and lower decile. Above the diagonal, the correlations between IL were in general positive and significant. The correlations between morphological traits were moderate to high, with the highest three correlations being those that relate IL_SIL with each of the other morphological traits, IL_HatW (0.55), IL_LofS (0.442) and IL_HofW (0.381). The correlation between the two morphological defects is negative and close to zero (− 0.065) and the correlations between the morphological traits and morphological defects are low, except for IL_HatW with IL_KK (positive and moderate value of 0.295) and with IL_CrN (negative and moderate value of − 0.257). The diagonal shows the percentage of males and females in the upper (animals with the highest IL) and the lower (animals with the lowest IL) decile compared to the total number of males (1662) and females (3364) in the reference population. In the upper decile, the percentage of males ranged from 10.8% (IL_HatW) to 12.5% (IL_CrN), whereas the percentage of females ranges from 8.8% (IL_LofS and IL_CrN) to 9.6% (IL_HatW). In the lower decile, the percentage of males ranged from 11.6% (IL_HofW and IL_KK) to 12.3% (IL_HatW) and the percentage of females ranged from 8.9% (IL_HatW) to 9.3% (IL_HofW). Under the diagonal in Table 6 are shown the percentages of coincident animals in the upper and lower decile of IL by pairs of traits. In the upper decile, the percentage of coincident animals ranged from 7% (IL_KK-IL_SIL) to 38% (IL_SIL-IL_HatW), whereas in the lower decile, the percentage of coincident animals ranged from 12% (IL_SIL-IL_KK and IL_SIL-IL_CrN) to 56% (IL_SIL-IL_HatW). The Pearson`s correlations between BV, the percentage of males and females in the upper and lower decile of BV compared to the reference PRE population, and the percentage of coincident animals between traits in the upper and lower decile were also calculated (see Additional file 2: Table S1).

**Table 6:** Inbreeding depression load genetic values: Pearson’s correlations and percentages of coincident animals (within and between traits)

	IL_HofW	IL_HatW	IL_LofS	IL_SIL	IL_KK	IL_CrN
IL_HofW	11.7%/9.2% (11.6%/9.3%)	0.292 ± 0.012*	0.226 ± 0.012*	0.381 ± 0.011*	–0.081 ± 0.015*	–0.109 ± 0.015*
IL_HatW	27% (46%)	10.8%/9.6% (12.3%/8.9%)	0.171 ± 0.013*	0.551 ± 0.009*	0.295 ± 0.012*	–0.257 ± 0.016*
IL_LofS	14% (34%)	21% (22%)	12.3%/8.8% (12.2%/9.0%)	0.442 ± 0.011*	0.049 ± 0.014*	0.084 ± 0.014*
IL_SIL	28% (44%)	38% (56%)	19% (41%)	11.4%/9.3% (12.0%/9.1%)	–0.003 ± 0.014	0.001 ± 0.014
IL_KK	11% (13%)	13% (20%)	8% (26%)	7% (12%)	11.0%/9.5% (11.6%/9.2%)	–0.065 ± 0.015*
IL_CrN	24% (17%)	12% (13%)	8% (24%)	17% (12%)	31% (23%)	12.5%/8.8 (12.2%/9.0%)

Above the diagonal: Pearson’s correlations ± SD between inbreeding depression load genetic values; on the diagonal: percentage of males/females animals in upper (lower) decile of inbreeding load in comparison with the reference population; under the diagonal: percentage of coincident animals in upper (lower) decile of inbreeding load.

*IL* Inbreeding load genetic value. *HofW* Height of withers , *HatW * Height at withers, *LofS* Length of shoulder, *SIL* Scapular-ischial length, *KK* Knock knee, *CrN *Cresty neck .

*Significant differences p < 0.05

## Discussion

Inbreeding depression has always been considered as a negative phenomenon, which decreases phenotypic values. However, there is overwhelming evidence that indicates that inbreeding depression is not homogeneous among the individuals of a population since it depends on the proportion of IBD alleles received from each ancestor. For this reason, several studies have detected heterogeneity in inbreeding depression for different traits among founder individuals in several species [7–11], and in some individuals it has been observed that the inbreeding value could even have a positive impact on phenotypic values [10]. Our study addresses, for the first time, the estimation of individual inbreeding depression load for morphological traits and defects in the horse, following the methodology recently proposed by Varona et al. [10]. To achieve this, we used the main Spanish horse breed as the reference population. This breed is characterized by its high inbreeding level, due to the closed breeding system applied by breeders.

Our results on the gene origin and genetic variability of the analysed population are in line with those obtained in previous studies [12, 13]. The increase in $F$ from generation 3 to 6 is consistent with the fact that the ancestor responsible for the inbreeding in the population originated from the first generations, and with the decrease in matings between relatives made by breeders in recent years in order to control the inbreeding rate. The mean $\Delta F$ value, which is a parameter that theoretically becomes constant and is independent of pedigree depth in an ideal population [25], can be attributed to the amount of partial inbreeding in the horses from which the pedigree was constructed. We found a $\Delta F$ of 1.33% in the reference population, which seems to be a low value, given the high $F$ level in the animals studied. As the PRE population is a closed population with a long and well-established studbook, the calculated parameters are reliable, as shown by the number of maximum, equivalent and complete generations. There is hardly no difference between the AR of animals in the total and active populations and that of the reference population (11.3% and 11.4% vs 10.8%). Since breeders tend to cross their animals, most of the inbred horses that are selected to conform to the reference population would be related mainly to other individuals of their own stud, but not very closely to those of the total population.

We assessed the effect of inbreeding on several traits by calculating the regression coefficients between ${F}_{3}$, ${F}_{6}$ and $F$, and Δ*F* and the phenotypic values of the population. Our results show the existence of significant inbreeding depression for all the morphological traits, regardless of the selection pressure that is applied on them, as regressions became more strongly negative or decreased over time. These results agree with the study by Gómez et al. [16], who reported similar effects of inbreeding on body measurements in the same breed. Moreover, such a reduction in phenotypic values for morphological traits and an increase in the prevalence of similar morphological defects, such as club foot, caused by increasing inbreeding have been demonstrated in other horse breeds [26, 27]. This negative relationship between $F$ and morphological traits and defects can be explained by the genetic load of partially deleterious alleles not being purged by natural or artificial selection in the breed, as shown by the standardized regression coefficient. Indeed, with the same increase in $F$, a larger decrease is observed for the morphological traits neutral to selection (HofW and SIL) than for morphological traits on which selection pressure is applied (HatW and LofS). In this case, the morphological traits and defects are neither harmful to health nor lethal (only animals with highly deleterious defects are disqualified), but they are more difficult to select out [1, 11]. Furthermore, Sanchez et al. [28] showed that one of the factors associated to CrN score is age, and that the scores are significantly higher for animals more than 6 years old than for younger ones. The fact that CrN appears at later ages and gets worse with time means that it is extremely difficult to eradicate before the horse reproduces. In addition, because of its low heritability, its prevalence does not increase in inbred animals. In fact, the $F$ of 89.9% of the horses that have been scored for CrN is lower than 12.5%, with most of the data on CrN being concentrated in low-inbred animals (see Additional file 3: Table S2).

Horse conformation is the result of a combination of natural and human selection for different breeders’ objectives. As well as beauty and breed standards, breeding animals are selected for body morphology to suit specific functions. In the PRE breed, the main objective is to improve functionality in dressage, including gait quality. While some body measurements remain neutral to selection, those that show a good correlation with performance are under intense selection [15].

Table 1 shows that there are differences in measurements (in cm) between males and females. Physical differences in body size associated with gender, known as sexual dimorphism, have previously been reported in this breed [15]. On the one hand, regarding the mean values of the morphological traits, our results are consistent with those of other studies, with stallions being on average taller and larger than mares [16, 24]. On the other hand, the scores for KK and CrN do not appear to be associated with sex in our reference population. KK is a defect of the joint that can affect both males and females in the same way. CrN is related to the accumulation of fat deposition in the dorsal neck and is usually related to sex but also to the horse’s age [29], which explains why we found no significance differences in our reference population, in which the average age of the horses with a neck defect is 4.9 years. This means that the horses have not yet had the opportunity to develop an adult hormonal pattern. The higher mean value for CrN than for KK means that CrN is a more frequent condition in this breed. Sánchez et al. [28] estimated the prevalence of CrN at 9% in the PRE population, which is high given that it is a disqualifying defect.

The inbreeding coefficient, $F$, has been widely used in animal breeding but the literature does not provide detailed information about the contribution of each founder to the probability of IBD. Some authors [19, 28] have described a limited interpretation of $F$, as an absolute measure of autozygosity, and that its value also depends on the depth of the pedigree. However, $F$ can be partitioned into coefficients attributed to specific founders, known as partial inbreeding coefficients, ${F}_{ij}$, the combined probability that an individual $i$ is autozygous (IBD) for an allele and that such an allele was derived from the allele in founder $j$ [30–32]. The Mendelian decomposition adaptation [17, 33], which divides an individual’s inbreeding according to the origin of coancestry of its parents, has allowed us to develop a methodology to predict the ${F}_{ij}$ of all the horses included in the pedigree file of PRE based on a mixed linear model [34]. The ${F}_{ij}$ inherent to a given individual is defined as the expected combined impact of all its inbreeding-related polymorphisms [8]. Within this framework, its inheritance pattern and the expected impact on fitness traits in the offspring of subsequent generations can be predicted [10]. Based on this methodology, we were able to estimate the variance components for the additive genetic effect and for the potential of inbreeding depression. Our results show, in all cases, that inbreeding depression load variance involves the larger part of the variance components (from one to several orders of magnitude). These differences are in line with the results of the study by Varona et al*.* [10], in which inbreeding depression load variances were larger than variance components, which is attributed to the wide range of predictions of inbreeding loads.

The estimates of heritability (Table 4) obtained in our study are very close to those previously reported [15]. Nonetheless, a higher value has been published for the heritability of CrN, 0.37 [28]. The inbreeding depression load ratios, obtained for a $F$ value of 10%, ranged from 0.04 (CrN) to 0.40 (LofS). This parameter is computed in the same way as for heritability and can be interpreted as the proportion of the variation of each trait in the population that is attributable to the variation in inbreeding depression load between individuals. While heritability is related to the variability expressed in the population, inbreeding depression load ratios measure the potential variability, whether it is expressed in the population or not. In fact, it cannot be expressed in its totality, because if an animal is 100% inbred with an ancestor, it cannot be fully inbred with other ancestors. In the case of HofW and LofS, the values of the inbreeding depression load surpass those of the heritability, which implies that the inbreeding depression weighed more in the explanation of the variability of these traits in a population where all the individuals have inherited 10% of their ${F}_{ij}$ from the same common ancestor. In addition, the fact that, HPD_95%_ due to the direct additive genetic effect and inbreeding depression load variances for HofW, KK and CrN include 0 can be attributed to their low heritability estimates. To date, the studies on inbreeding depression load ratios [5, 10] have reported low ratios for the inbreeding depression load, which added to the wide variability of this parameter, determine very wide confidence limits. Finally, our results show that there is no relationship between the variables submitted to more or less selection pressure and the heritability and inbreeding depression load ratios of the traits. Nevertheless, the PRE breeding program was initiated in 2003, almost two generations ago, which means that the number of generations is not yet sufficient to observe differences between traits on which selection pressure has been applied or not.

Analysis of the evolution in average inbreeding depression loads in the reference PRE population across generations (Fig. 1) shows that the variation is not the same for all the traits and that it does not follow a clear pattern. In general, the average negative inbreeding depression load values become more strongly negative, whereas the average positive inbreeding depression load values remain constant or increase. The positive values do not increase as much as the negative values decrease, thus, on average, inbreeding depression load values become more negative for all traits. A negative inbreeding depression load means that, for a horse with a sufficiently high partial inbreeding coefficient that derives from a certain ancestor, the magnitude of the morphological trait value will decrease and defects will get worse. In other words, animals with more negative inbreeding depression load values will have inbred descendants with a worse phenotype. Overall, across the last few generations, the morphological traits of the descendants with high and negative partial inbreeding values will have lower average records for HofW, HatW, LofS and SIL, and the prevalence of KK and CrN will increase. In addition, an explanation for the negative inbreeding depression load values becoming more negative across generations, could be that, with time, the frequency of deleterious mutations for the morphological traits and defects derived from certain ancestors, may have increased along with their genetic contribution to the population. Parallel to that, from 1934 to 1963 and especially between 1994 and 2003, the average inbreeding depression loads for CrN stand out with conspicuously positive values. One plausible hypothesis is that this condition, which is disqualifying in the breed standard, was associated with a grey coat colour [28] and animals with this coat colour were partially avoided as breeding animals. Indeed, some years ago, the breeder’s preferences for coat colour of the PRE breed changed drastically from the usual grey to less common colours (chestnut, bay, pearl, etc.), although grey is again fashionable in recent years [23].

The upward trend of the classical $F$ is accompanied by the largest increase in negative inbreeding depression loads values. This fits with the deeply-rooted idea that inbreeding has deleterious effects: higher values of inbreeding are accompanied by potentially worse phenotypes in the offspring. However, as mentioned before, even when the average inbreeding depression load values for traits and defects are negative, some animals in the population show positive values. Thus, an average negative inbreeding depression load for a trait does not imply that the morphology of all the inbred offspring will get worse. A purging effect may have occurred in some lineages, such that the most extreme individuals have been eliminated across generations and the number of animals with positive values increased, with certain lineages and traits being more selected than others.

Related animals can be mated with the aim of increasing or decreasing a certain character or defect and the result depends on the partial inbreeding and inbreeding depression load of the common ancestors. In this way, the correlations observed between inbreeding depression load genetic values and breeding values are of great interest. In Table 5, the Pearson’s correlations between additive and inbreeding depression load genetic values for the same trait are on the diagonal. They are all positive except for SIL, which is almost zero, and for KK and CrN. Positive correlations imply that horses with higher breeding values cause a particularly positive inbreeding effect in their inbred descendants. Regarding HofW, which presents a correlation of 0.67, an animal with a high breeding value for this trait will transmit longer height of withers to its descendants, especially those that have received high partial inbreeding from these ancestors. In contrast, the negative correlations between additive genetic and inbreeding depression effects for CrN and KK, suggest that the selection of animals with high genetic values for these defects would result in some negative effects due to inbreeding depression if the inbreeding depression load values of the most influential ancestors in the population are not taken into account. In the case of defects, the more negative the inbreeding depression load of an animal is, the more positive is the breeding value and, thus, in terms of effects, the higher becomes the prevalence of the defect in a potential inbred descendant.

The Pearson’s correlations between additive and inbreeding depression load genetic values for the different traits (off-diagonal values in Table 5) are positive and/or near null, which suggests that individuals with high genetic values for morphological traits will not cause much inbreeding depression, even if for some traits the correlations were negative. However, it is very important to take into account that the selection of horses based on their high additive genetic values for one morphological trait could lead to different inbreeding depression loads for the rest of morphological traits.

The Pearson’s correlations between inbreeding depression load genetic values for the different traits are above the diagonal in Table 6. Most of these values are positive or near zero, except the correlations of CrN with HofW and HatW, which are negative and low to moderate, respectively. Positive correlations indicate that horses with a higher inbreeding depression load in the genetic value for given morphological trait would also have a positive inbreeding effect on their inbred descendants for the other trait. Conversely, negative correlations imply that an animal with a higher inbreeding depression load genetic value for a given morphological trait would lead to a negative inbreeding effect in their inbred descendants for the other trait. Therefore, according to our results, exerting selection pressure on horses with positive and high genetic values of inbreeding depression load will have, in general, positive effects for most traits. Thus, it can be inferred that most of the traits and defects evaluated in this work are genetically related and are similarly affected under inbreeding.

On the diagonal in Table 6, the percentages of males and females in the upper and lower deciles compared to the total number of males and females in the reference population, range from 8.8 to 12.5% (i.e. around 10%), which indicates that the inbreeding depression load genetic value is almost equally distributed between genders, it is only slightly higher in males. However, the percentages of coincident animals in the upper and lower deciles between different traits (under the diagonal) show more differences. The percentages of coincident animals in the lower decile are, in general, higher than the percentages of coincident animals in the upper decile. Considering that, for horses from the same generation, the degree of pedigree information will be the same and therefore the estimates for each one will move away from zero, this implies that animals with the lowest inbreeding depression load genetic values for one trait also tend to have the lowest inbreeding depression load genetic values for the other traits. These results also corroborate the correlations between inbreeding depression load genetic values, which show the strongest coincidences between traits with the highest correlations and vice versa. In spite of this information, an efficient management of inbreeding levels will continue to be an important goal for breeding programs in horse breeds such as PRE, in order to ensure that populations can adapt to future breeding goals while avoiding the accumulation of detrimental effects associated with this inbreeding.

Finally, genomic analyses can improve the knowledge about the regions that contribute this negative genetic load [35]. The results of the genomic analyses, in addition to presenting a greater understanding of the genomic bases of inbreeding depression, may play an important role in the genetic management of the breeding program. Region-specific knowledge should allow breeders to manage the risks associated with breeding animals or selection of mates more effectively, and to better evaluate the trade-off between the genetic value of the progeny and the undesirable side effects associated with inbreeding [36]. Thus, mating a stallion with potential mares that have similar inbreeding values might be more effective based on haplotype differences. In this context, the kind of analyses that we carried out in this work could prove to be of great use for the selection of animals to be genotyped.

## Conclusions

Our findings demonstrate that taking the potential of inbreeding depression carried by the future breeders into account is important and that the result of mating related animals with each other for increasing or decreasing a trait or defect not only depends on their additive genetic value, but also on their partial inbreeding and the inbreeding depression load genetic value of the common ancestor. Thus, the knowledge of the individual inbreeding depression load genetic values of stallions and mares for a particular trait is of great interest to the PRE Breeding Programme and could be used to guide the choice of breeding animals. The possibility of using animals with a high genetic value for the selection criteria which, in turn, were less prone to manifesting deleterious effects of inbreeding, could lead to a more favourable, persistent response to selection.

## Supplementary information

**Additional file 1: Figure S1.** Representation of the four morphological traits analysed in PRE horses. *HofW* Height of withers, *HatW* Height at withers, *SIL* Scapular-ischial length, *LofS* Length of shoulder

**Additional file 2: Table S1.** Breeding values: Pearson’s correlations and percentages of coincident animals (within and between traits). Above the diagonal: Pearson’s correlations ± SD between breeding values; on the diagonal: percentage of males/females animals in upper (lower) decile of breeding values in comparison with the reference population; under the diagonal: percentage of coincident animals in upper (lower) decile of breeding values. *BV* Breeding value, *HofW* Height of withers, *HatW* Height at withers, *LofS *Length of shoulder, *SIL* Scapular-ischial length, *KK* Knock knee, *CrN* Cresty neck.

**Additional file 3: Table S2. ** Percentage of animals affected by different classes of the studied defects and mean inbreeding values. N(%) percentage of animals, F(%) average inbreeding values, in percentage.

## Data Availability

The dataset supporting the results of this study was supplied by the National Association of Pura Raza Española Horse Breeders (ANCCE). The datasets generated and/or analysed during the current study are available from the corresponding author upon reasonable request.
