# Supplementary material for: Genetic inbreeding depression load for morphological traits and defects in the Pura Raza Española horse
Source: Genet Sel Evol. 2020 Oct 20;52:62. doi: 10.1186/s12711-020-00582-2 (PMC7576714; doi:10.1186/s12711-020-00582-2)
Supplement: Supplementary file 1 — Additional file 1: Figure S1. Representation of the four morphological traits analysed in PRE horses. HofW Height of withers, HatW Height at withers, SIL Scapular-ischial length, LofS Length of shoulder [file 12711_2020_582_MOESM1_ESM.docx]

Format: .doc

Title: Representation of the four morphological traits analysed in PRE horses.

Description: HofW: Height of withers; HatW: Height at withers; SIL: Scapular-ischial length; LofS: Length of shoulder.


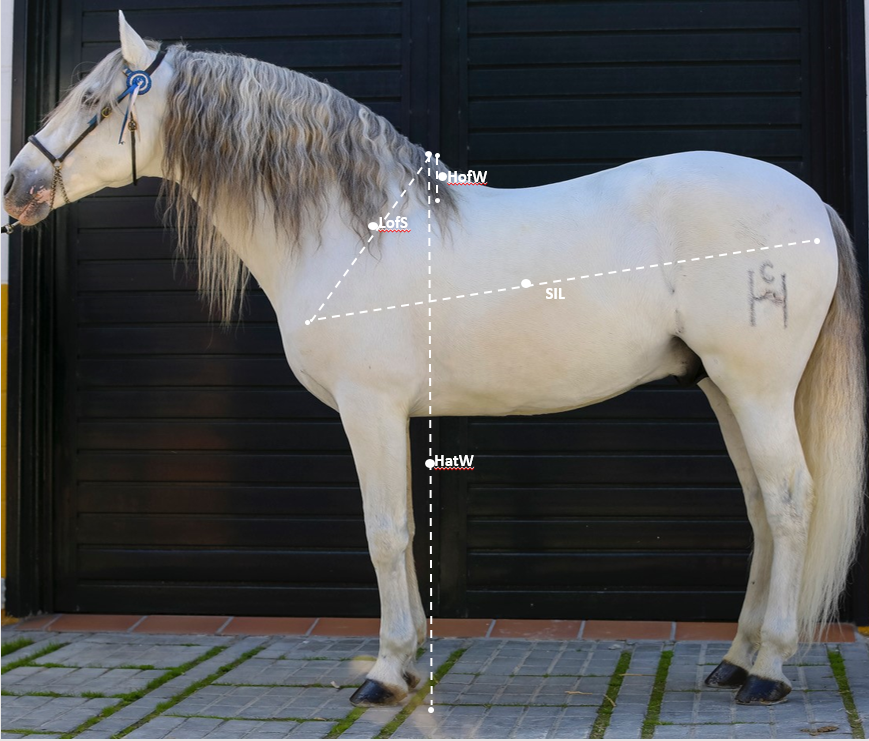


- *Height of withers*: distance, measured in centimetres, between the height at withers and the height at lowest point of withers.
- *Scapular-ischial length*: distance, measured in centimetres, of the straight segment from the shoulder joint to the point of buttock.
- *Height at withers*: distance, measured in centimetres, from the ground to the ridge between the shoulder blades.
- *Length of shoulder*: distance, measured in centimetres, from the point of the shoulder to the highest point of the withers.

In addition, following the ANCCE basic aptitude assessment sheet, the defects studied are coded as:

- *Knock knee*: condition in which the knees are pointing outwards instead of being vertically aligned with the forehands. This was measured on a scale of 1 - 5, where 1 is a vertically-aligned knee, 3 is slightly deviated and 5 is noticeably deviated.
  - Class 1: Without defect
  - Class 2 and 3: Slight defect but approved
  - Class 4: Serious defect
  - Class 5: Very serious defect
- *Cresty neck*: condition in which the neck accumulates fat deposits, looks wider and eventually droops to one side. This was measured on a scale of 1 - 9, where 1 is a total absence of the defect, 5 an incipient defect and 9 when the crest permanently droops to one side.
  - Class 1: Without defect
  - Class 2, 3 and 4: Slight defect but approved
  - Class 5: Serious defects
  - Class 6: Very serious defect
  - Class 7, 8 and 9: Disqualifying defect, horses have difficulties of movement
