# Supplementary material for: Genetic inbreeding depression load for morphological traits and defects in the Pura Raza Española horse
Source: Genet Sel Evol. 2020 Oct 20;52:62. doi: 10.1186/s12711-020-00582-2 (PMC7576714; doi:10.1186/s12711-020-00582-2)
Supplement: Supplementary file 2 — Additional file 2: Table S1. Breeding values: Pearson’s correlations and percentages of coincident animals (within and between traits). Above the diagonal: Pearson’s correlations ± SD between breeding values; on the diagonal: percentage of males/females animals in upper (lower) decile of breeding values in comparison with the reference population; under the diagonal: percentage of coincident animals in upper (lower) decile of breeding values. BV Breeding value, HofW Height of withers, HatW Height at withers, LofS Length of shoulder, SIL Scapular-ischial length, KK Knock knee, CrN Cresty neck. [file 12711_2020_582_MOESM2_ESM.docx]

Format: .doc

Title: Breeding values: Pearson’s correlations and percentages of coincident animals (within and between traits).

Description: Above the diagonal: Pearson’s correlations ± SD between breeding values; on the diagonal: percentage of males/females animals in upper (lower) decile of breeding values in comparison with the reference population; under the diagonal: percentage of coincident animals in upper (lower) decile of breeding values. BV: Breeding value. Height of withers (HofW); Height at withers (HatW); Length of shoulder (LofS); Scapular-ischial length (SIL); Knock knee (KK); Cresty neck (CrN).

|  | **BV_HofW** | **BV_HatW** | **BV_LofS** | **BV_SIL** | **BV_KK** | **BV_CrN** |
| --- | --- | --- | --- | --- | --- | --- |
| **BV_HofW** | *10.7%/9.7%*  *(11.0%/9.5%)* | 0.165±0.014* | 0.090±0.014* | 0.009±0.014 | -0.079±0.014* | -0.075±0.014* |
| **BV_HatW** | 29%  (32%) | *10.8%/9.6%*  *(11.7%/9.2%)* | 0.540±0.012* | 0.490±0.012* | 0.105±0.014* | -0.059±0.014* |
| **BV_LofS** | 21%  (30%) | 44%  (53%) | *11.0%/9.5%*  *(11.9%/9.1%)* | 0.340±0.013* | 0.128±0.014* | 0.089±0.014* |
| **BV_SIL** | 21%  (12%) | 48%  (26%) | 39%  (23%) | *11.5%/9.3%*  *(10.1%/10.0%)* | -0.098±0.014* | -0.133±0.014* |
| **BV_KK** | 12%  (13%) | 18%  (26%) | 15%  (29%) | 14%  (9%) | *11.0%/9.6%*  *(8.4%/9.2%)* | 0.132±0.014* |
| **BV_CrN** | 15%  (14%) | 15%  (15%) | 17%  (26%) | 14%  (7%) | 26%  (25%) | *11.1%/9.5%*  *(12.0%/9.1%)* |

^*^Significant differences p<0.05.
