# Supplementary material for: Genetic inbreeding depression load for morphological traits and defects in the Pura Raza Española horse
Source: Genet Sel Evol. 2020 Oct 20;52:62. doi: 10.1186/s12711-020-00582-2 (PMC7576714; doi:10.1186/s12711-020-00582-2)
Supplement: Supplementary file 3 — Additional file 3: Table S2. Percentage of animals affected by different classes of the studied defects and mean inbreeding values. N(%) percentage of animals, F(%) average inbreeding values, in percentage. [file 12711_2020_582_MOESM3_ESM.docx]

|  |  |  | **Without defect**  **(class 1)**  ***, **** | **Approved**  **(class 2-4)***  **(class 2-3)**** | **Serious**  **Defect**  **(class 5)***  **(Class 4)**** | **Very serious defect**  **(class 6)***  **(class 5)**** | **Disqualified**  **(class 7-9)*** |
| --- | --- | --- | --- | --- | --- | --- | --- |
| **Knock knee** | **N (%)** | | 67.64 | 32.03 | 0.28 | 0.01 | **-** |
|  | **F (%)** | | 7.37 | 7.42 | 7.52 | 14.46 | **-** |
| **Cresty neck** | **N (%)** | | 78.19 | 16.64 | 4.16 | 0.63 | 0.38 |
|  | **F (%)** | | 7.39 | 7.40 | 7.40 | 7.25 | 8.12 |

Format: .doc

Title: Percentage of animals affected by different classes of the defects studied and mean inbreeding values.

Description: N(%): percentage of animals, F(%): average inbreeding values, in percentages.

*Cresty neck; **Knock knee.
